# Cell Turnover and Detritus Production in Marine Sponges from Tropical and Temperate Benthic Ecosystems

**DOI:** 10.1371/journal.pone.0109486

**Published:** 2014-10-07

**Authors:** Brittany E. Alexander, Kevin Liebrand, Ronald Osinga, Harm G. van der Geest, Wim Admiraal, Jack P. M. Cleutjens, Bert Schutte, Fons Verheyen, Marta Ribes, Emiel van Loon, Jasper M. de Goeij

**Affiliations:** 1 Department of Aquatic Ecology and Ecotoxicology, Institute for Biodiversity and Ecosystem Dynamics, University of Amsterdam, Amsterdam, The Netherlands; 2 Porifarma B.V. Poelbos 3, Ede, The Netherlands; 3 Department of Pathology, Cardiovascular Research Institute Maastricht, Maastricht University, Maastricht, The Netherlands; 4 Department of Molecular Cell Biology, Research Institute Growth and Development, Maastricht University, Maastricht, The Netherlands; 5 Electron Microscopy Unit, CRISP, Maastricht, The Netherlands; 6 Institut de Ciències del Mar-Consejo Superior de Investigaciones Científicas (ICM-CSIC), Barcelona, Spain; 7 Department of Computational Geo-Ecology, Institute for Biodiversity and Ecosystem Dynamics, University of Amsterdam, Amsterdam, The Netherlands; Australian Institute of Marine Science, Australia

## Abstract

This study describes *in vivo* cell turnover (the balance between cell proliferation and cell loss) in eight marine sponge species from tropical coral reef, mangrove and temperate Mediterranean reef ecosystems. Cell proliferation was determined through the incorporation of 5-bromo-2′-deoxyuridine (BrdU) and measuring the percentage of BrdU-positive cells after 6 h of continuous labeling (10 h for *Chondrosia reniformis*). Apoptosis was identified using an antibody against active caspase-3. Cell loss through shedding was studied quantitatively by collecting and weighing sponge-expelled detritus and qualitatively by light microscopy of sponge tissue and detritus. All species investigated displayed substantial cell proliferation, predominantly in the choanoderm, but also in the mesohyl. The majority of coral reef species (five) showed between 16.1±15.9% and 19.0±2.0% choanocyte proliferation (mean±SD) after 6 h and the Mediterranean species, *C. reniformis*, showed 16.6±3.2% after 10 h BrdU-labeling. *Monanchora arbuscula* showed lower choanocyte proliferation (8.1±3.7%), whereas the mangrove species *Mycale microsigmatosa* showed relatively higher levels of choanocyte proliferation (70.5±6.6%). Choanocyte proliferation in *Haliclona vansoesti* was variable (2.8–73.1%). Apoptosis was negligible and not the primary mechanism of cell loss involved in cell turnover. All species investigated produced significant amounts of detritus (2.5–18% detritus bodyweight^−1^·d^−1^) and cell shedding was observed in seven out of eight species. The amount of shed cells observed in histological sections may be related to differences in residence time of detritus within canals. Detritus production could not be directly linked to cell shedding due to the degraded nature of expelled cellular debris. We have demonstrated that under steady-state conditions, cell turnover through cell proliferation and cell shedding are common processes to maintain tissue homeostasis in a variety of sponge species from different ecosystems. Cell turnover is hypothesized to be the main underlying mechanism producing sponge-derived detritus, a major trophic resource transferred through sponges in benthic ecosystems, such as coral reefs.

## Introduction

Sponges are key components of aquatic ecosystems. On coral reefs, a large proportion of the available suspended [1] and dissolved [2] organic energy and nutrients are retained by sponges and subsequently transferred to higher trophic levels through the so-called ‘sponge loop’ [3]. When including dissolved organic matter (DOM) in the energy budgets of sponges, the majority (81–95%) of the daily diet of investigated sponges on coral reefs consists of DOM [2], [4], [5]. The conversion of DOM into particulate organic matter (POM, also referred to as detritus) through rapid proliferation and shedding of sponge cells is proposed to be the main underlying mechanism involved in the transfer of DOM to higher trophic levels [3]. This proposition is based on the study of cell turnover in a single species of tropical coral reef sponge, *Halisarca caerulea*. The filter cells (choanocytes) of *H. caerulea* proliferate rapidly, with a cell cycle duration of only 5.4 h, one of the fastest described to date in any multi-cellular animal *in vivo* [6]. Under steady-state, i.e. non-growing conditions, rapid cell proliferation is balanced with massive amounts of cell shedding in order to maintain tissue homeostasis in the choanocyte compartment. The ‘old’ choanocytes are thought to be expelled as detritus into the water column [6]. Tissue turnover is evident in *H. caerulea* and in three additional coral reef sponge species; *Chondrilla caribensis, Scopalina ruetzleri* and *Haliclona implexiformis* (now re-identified as *Haliclona vansoesti*), which have been shown to convert assimilated ^13^C- and ^15^N-enriched DOM into ^13^C- and ^15^N-enriched detritus [3]. However, cell proliferation and shedding have not yet been investigated in species other than *H. caerulea*.

Sponges are widely recognized as one of the earliest metazoan life forms, e.g. [7], [8]. However, little is known about tissue homeostasis in sponges, which is one of the basal prerequisites of multi-cellularity. Tissue homeostasis is the regulation of cell proliferation and cell loss, also known as cell turnover, which acts to maintain a healthy population of cells. Cell turnover occurs in many fully developed metazoan tissues and keeps tissues in a constant state of cellular flux. This is mediated through the elimination of differentiated cells that are replaced by the proliferation of adult stem cells [9], [10]. Sponges display slow growth rates both *in situ* and *ex situ* [11], however, they have potential for rapid proliferation as demonstrated by their ability to grow new tissue at up to 2,900 times their normal growth rate during tissue regeneration in response to wound infliction [12]. Division of choanocytes by mitosis has previously been reported in a few sponge species, including the marine sponge *Hymeniacidon sinapium* [13] and the freshwater sponge *Ephydatia fluviatilis* [14].

Apoptosis (programmed cell death) and cell shedding are widely regarded as common mechanisms of cell loss involved in the homeostasis of metazoan tissues [10], [15]. Apoptotic pathways have been identified in sponges, e.g. cDNA's encoding predicted caspase-3-related proteins have been isolated from specimens of *Geodia cydonium* and *Suberites domuncula* [16] and apoptosis involving caspase-3 has been measured in sponge cell assays [17]. Additionally, anti-active caspase-3 antibodies have been used to detect caspase-3 activity in sponge tissue sections [6], [18]. However, only low amounts of apoptosis have been found in *H. caerulea*, located predominantly in the mesohyl compartment and not in the proliferative choanoderm [6]. Apoptosis is therefore not considered to be the principle mechanism of cell loss involved in cell turnover under steady-state conditions for this species. The role of apoptosis in cell turnover is unknown for other sponge species.

Cell shedding, as described in *H. caerulea* [6], is a common mechanism of cell loss for the maintenance of tissue homeostasis in highly proliferative tissues, such as the epithelium of the mammalian gastrointestinal tract, e.g. [19]–[21]. Evidence for cell shedding in other sponge species is still lacking, but the link between sponge biomass turnover and the production of sponge-derived detritus is becoming more evident. Direct evidence shows the production of significant amounts of detritus as a result of dissolved organic matter assimilation in four species of tropical coral reef sponge [3]. Detritus production has also been observed in a variety of additional tropical marine sponges [4], [22] and in sponges from other marine ecosystems, such as deep-sea cold water [23], Mediterranean [24] and freshwater ecosystems [25]. However, this detritus production has generally been linked to feeding waste, and cellular debris derived from cell turnover has not been taken into account.

Sponges have abundant and diverse communities of associated microbes in their tissues, e.g. [26], [27], and species are generally categorized as either low microbial abundance (LMA) sponges, which have microbial abundances similar to seawater, or high microbial abundance (HMA) sponges, with microbial concentrations 2–4 orders of magnitude higher than seawater [28]–[30]. According to genomic analysis, sponge-associated microbes should be capable of a broad range of metabolic transformations [27]. The question is whether these microbes in turn affect tissue homeostasis of the sponge holobiont.

We hypothesize that cell turnover through cell proliferation (predominantly in choanocytes) and cell shedding is a general mechanism for maintaining tissue homeostasis under steady-state conditions in a wide variety of sponge species from various benthic marine ecosystems. We therefore investigated *in vivo* cell proliferation, cell loss (through cell shedding and apoptosis) and detritus production in eight sponge species, with different abundances of associated microbes, from tropical coral reef, mangrove, and temperate Mediterranean reef ecosystems. Cell proliferation in sponge tissue was investigated by *in vivo* labeling with the thymidine-analogue 5-bromo-2′-deoxyuridine (BrdU) and subsequent immunohistochemical staining of tissue sections. Cell loss through apoptosis was investigated by immunohistochemistry using an antibody against active caspase-3. Cell loss through shedding was assessed qualitatively in histological sections and the dry weight of detritus produced daily by sponges was determined.

## Materials and Methods

### Ethics statement

Research on Curaçao was performed under the research permit (#2012/48584) issued by the Curaçaoan Ministry of Health, Environment and Nature (GMN) to the CARMABI foundation.

### Sponge species and collection

We studied eight demosponge (Porifera: Demospongiae) species; six tropical coral reef species (Halisarca caerulea, Chondrilla caribensis, Scopalina ruetzleri, Clathria sp., Haliclona vansoesti and Monanchora arbuscula), one mangrove species (Mycale microsigmatosa) and one temperate Mediterranean reef species (Chondrosia reniformis). Tropical reef and mangrove species were collected by SCUBA diving or snorkeling on the reefs of the Caribbean island of Curaçao (12^°^12′N, 68^°^56′W), between February and April 2011 and 2013. The Mediterranean reef species was collected at the Medes Islands, Catalunya, Spain (42^°^05′N, 3^°^23′W) between August and September 2011. Sponges were chiseled from the (coral) rock or mangrove root and collected attached to their substrate, which was cleared of other organisms. All sponges were trimmed to a size of approximately 25 cm^2^ with no available substrate for growth in order to induce steady-state conditions. Specimens were kept in 100 L running seawater aquaria with a flow rate of 3 L min^−1^ (exchange rate of 33 min) at ambient temperature (26–27°C for tropical aquaria and 18–20°C for temperate aquaria). Sponges were allowed to acclimatize for a minimum of one week prior to incubation experiments. Any changes in the shape and size of sponges were noted during the experimental period of up to 4 weeks in order to ensure steady-state conditions.

### BrdU-labeling, fixation and embedding

Individual sponges (n = 3 per species, *H. vansoesti* n = 8) were enclosed in incubation chambers (3 L) with magnetic stirring devices [3], [6]. Incubation chambers were kept in the aquaria to maintain ambient seawater temperature. In order to measure cell proliferation, 5-bromo-2′-deoxyuridine (BrdU, Sigma) was added to incubation chambers containing the sponges. Sponges were incubated in seawater containing 50 µmol L^−1^ BrdU (optimal BrdU concentration was determined during preliminary tests) for 6 h (continuous labeling) in order to estimate and compare the relative amount of cell proliferation between numerous species and to test if the choanoderm is the predominant location of proliferating cells. According to the one population model [31], BrdU-labeling over time results in a linear increase in the number of BrdU-positive cells until the growth fraction is reached, after which there is no further increase in the number of BrdU-positive cells, (see also [6]). The length of the cell cycle is determined by the length of the linear increase and the growth fraction indicates the proportion of cells actively involved in proliferation. After 6 h of BrdU-labeling, choanocytes have either reached their growth fraction, or not, in which case the number of BrdU-positive choanocytes is still increasing linearly with time. If after 6 h of BrdU-labeling choanocytes have not yet reached their growth fraction, the number of cells labeled gives an indication of the rate of choanocyte proliferation, i.e. the number of choanocytes replaced every 6 h. Based on the 5.4 h cell cycle in *H. caerulea* [6], we assume that the cell cycle duration of choanocytes in other sponge species under steady-state conditions is unlikely to be much shorter than 6 h. If the cell cycle is shorter than 6 h, proliferation rates given here are slightly underestimated. For specimens of *C. reniformis* we were only able to access data from 10 h BrdU-incubations. Additional specimens of *H. caerulea* were incubated with BrdU for 10 h to determine if there are differences in the percentage of BrdU-positive cells between 6 h and 10 h of labeling in this species, i.e. to determine whether the growth fraction for choanocytes has been reached.

Immediately after the incubations, sponge tissue samples (∼0.5 cm^2^) were fixed in 4% paraformaldehyde in phosphate-buffered saline (PFA/PBS; 4 h at 4°C), rinsed in PBS, dehydrated through a graded series of ethanol and stored in 70% ethanol at 4°C until further processing. For histological investigations of *in situ* shedding, sponge tissue samples (∼1 cm^2^) were collected from all species (n = 3 per species) and placed in 15 mL syringes *in situ* (underwater) with the plunger removed. Seawater was pushed out using the plunger and a second syringe was used to transfer 4% PFA/PBS to the syringe containing sponge tissue, also performed *in situ* (underwater). Samples were transported to the lab within 2 h and post-fixed in 4% PFA/PBS (4 h at 4°C), rinsed in PBS, dehydrated through a graded series of ethanol and stored in 70% ethanol at 4°C until further processing. All tissue samples were embedded in butyl-methyl-methacrylate (BMM).

### BrdU-immunohistochemistry

Semi-thin sections (2 µm) of the embedded sponge tissue were cut on an ultramicrotome (Leica EM UC6) using a diamond knife (dEYEmond Histo 5 mm, Isselburg, Germany) and collected on glass slides (StarFrost, Knittelglass). BMM was removed in acetone and endogenous peroxidase activity was blocked by incubating slides in methanol containing 0.3% H_2_0_2_ (20 min). Slides were washed in tris-buffered saline (TBS) and incubated in citric acid (0.2% pH 6.0, 30 min at 85°C). After subsequent washing in TBS, DNA was denatured in HCl (2 mol L^−1^, 30 min at 37°C), pH-neutralized in sodium borate buffer (pH 8.5), and washed with TBS. Slides were incubated with mouse anti-BrdU monoclonal antibody (Nordic-MUbio MUB0200S, 1∶50 in TBS with 1% BSA, 0.1% Tween 20, 60 min) then washed in TBS. Primary antibody was detected using an avidin-biotin enzyme complex (Vectastain Elite ABC Kit, Vector laboratories). Slides were incubated with biotinylated rabbit anti-mouse antibody (in TBS with 1% BSA, 0.1% Tween 20, 30 min), washed in TBS and then incubated in avidin-biotin-peroxidase complex (in TBS with 1% BSA, 0.1% Tween 20, 30 min). Peroxidase activity was visualized with DAB (DAKO; 5–10 min; positive cells have brown-stained nuclei). Sections were washed in distilled water then counterstained in haematoxylin to visualize BrdU-negative nuclei (stained dark blue), dehydrated through a graded series of ethanol and mounted in Entellan (Merck). BrdU-labelled mouse intestinal tissue was used as a positive control and immunohistochemistry without primary antibody (on both mouse and sponge tissue) served as a negative control.

### Analysis of cell proliferation

All slides were examined under a light microscope (Olympus BH-2) and photographs were taken using an Olympus DP70 camera. To quantify the number of proliferative cells per species, two tissue samples were taken per specimen (middle section with and without osculum). From each tissue sample three areas of the sponge were sectioned, each approximately 100 µm apart. The percentage of BrdU-positive cells in the choanoderm (choanocytes) and in the mesohyl (all cells located between the pinacoderm and the choanoderm) was calculated per section. At least 250 choanocytes and 250 mesohyl cells were counted from each section making a total of at least 1500 (2 tissue areas x 3 sections x 250 cells) of each cell type counted per sponge.

### Cell shedding, detritus collection and quantification

Cell shedding was assessed qualitatively using light microscopy to observe the presence of shed cells in histological sections of sponge tissue sampled *in situ*. To quantify the production of detritus by sponges, five species were selected; *H. caerulea* (n = 7), *C. caribensis* (n = 5), *S. ruetzleri* (n = 8), *H. vansoesti* (n = 5), and *M. arbuscula* (n = 2). Sponges were placed in individual open-topped plastic pots (9.4 cm x 4.2 cm) within the aquaria and detritus was collected every 24 h for up to six consecutive days from the bottom surface of the pots and from the surface of the sponge using a glass Pasteur pipette. Detritus was centrifuged (340 x g, 5 min) and the supernatant discarded. Samples were freeze-dried overnight. The sponges were removed from their substrates and oven-dried for 24 h at 60°C. Dry-weights (DW) of both detritus and sponge tissue were determined. Pots without sponge (n = 3) served as controls from which detritus was collected for six consecutive days. The average percentage body weight (DW detritus DW sponge ^−1^) produced as detritus over 24 h was calculated for each of the five species tested. Additionally, detritus samples from *H. caerulea* (n = 5) were fixed in 4% PFA for 4 h for light microscopy analysis. Samples were rinsed in PBS, dehydrated through a graded series of ethanol and stored in 70% ethanol until further processing. Samples were embedded in gelatin prior to paraffin embedding and sectioning (5 µm). Sections were stained with haematoxylin and viewed using a light microscope in order to identify cellular debris within the detritus.

### Active caspase-3 immunohistochemistry and analysis of tissue sections

After removing BMM and blocking endogenous peroxidase activity, slides were washed in TBS and incubated in citric acid (see BrdU-immunohistochemistry). Slides were washed in TBS and blocked with goat serum (2% in TBS, 0.1% Tween 20, 30 min). After washing in TBS, slides were incubated with rabbit anti-active caspase-3 monoclonal antibody (BD Pharmingen, 1∶200 in TBS with 1% BSA, 0.1% Tween 20, 60 min). Slides were washed in TBS, then incubated with biotinylated goat anti-rabbit antibody (Vectastain Elite ABC Kit, Vector Laboratories; in TBS with 1% BSA, 0.1% Tween 20, 30 min), washed in TBS and then incubated in avidin-biotin-peroxidase complex. Peroxidase activity was visualized with DAB (active caspase-3 positive cells contain brown stained cytoplasm). Slides were counterstained in haematoxylin, dehydrated through a graded series of ethanol and mounted in Entellan. Human tonsil tissue served as a positive control and immunohistochemistry without primary antibody (on both human and sponge tissue) served as a negative control. The percentages of active caspase-3 positive cells were counted in at least 3 tissue sections per species, each from a different sponge individual. At least 250 mesohyl cells were counted per section.

### Sponge-associated microbial abundance classification using transmission electron microscopy (TEM)

Samples were fixed in 2.5% glutaraldehyde, 0.1 mol L^−1^ cacodylic acid in 0.2 µm-filtered seawater for 1 h at room temperature. Samples were post-fixed in 1% osmium tetroxide (OsO_4_) in filtered seawater for 1 h, dehydrated in a graded series of ethanol and stored in 70% ethanol until further processing. Tissue was embedded in epon-araldite. TEM images were used to categorize sponges as high microbial abundance (HMA) or low microbial abundance (LMA) as done by Hentschel and colleagues [30].

### Statistical analysis

In order to test if the choanoderm is the predominant location of proliferating cells compared to the mesohyl, the difference between the percentages of proliferating choanocytes versus mesohyl cells was investigated. This was done as a function of different variables: species identity, observed cell shedding (high or low) and tissue area (middle with or without osculum). For this purpose a linear mixed model was used [32], using the individual organism as a random effect. To determine the effects of these models, the p-values are reported, based on an F-test with a Kenward-Roger df approximation [33], which compares the full model with a model involving only a random effect. In addition, the 95%-confidence intervals (95%-CI) of the model coefficients are reported for the significant models. Additionally, a linear mixed model was used to test the difference in the percentage of BrdU-positive choanocytes between 6 h and 10 h of continuous labeling in *H. caerulea*. A linear mixed model was also used to investigate the difference in detritus production between test pots with sponge and control pots without sponge, using the individual organism as a random effect. The relationship between the percentage detritus biomass^−1^ d^−1^ and observed cell shedding (high or low) was investigated using a linear model. All calculations were conducted in R, using the lme4 package (R Development Core Team 2013).

## Results

### Cell proliferation

**Figure 1:**
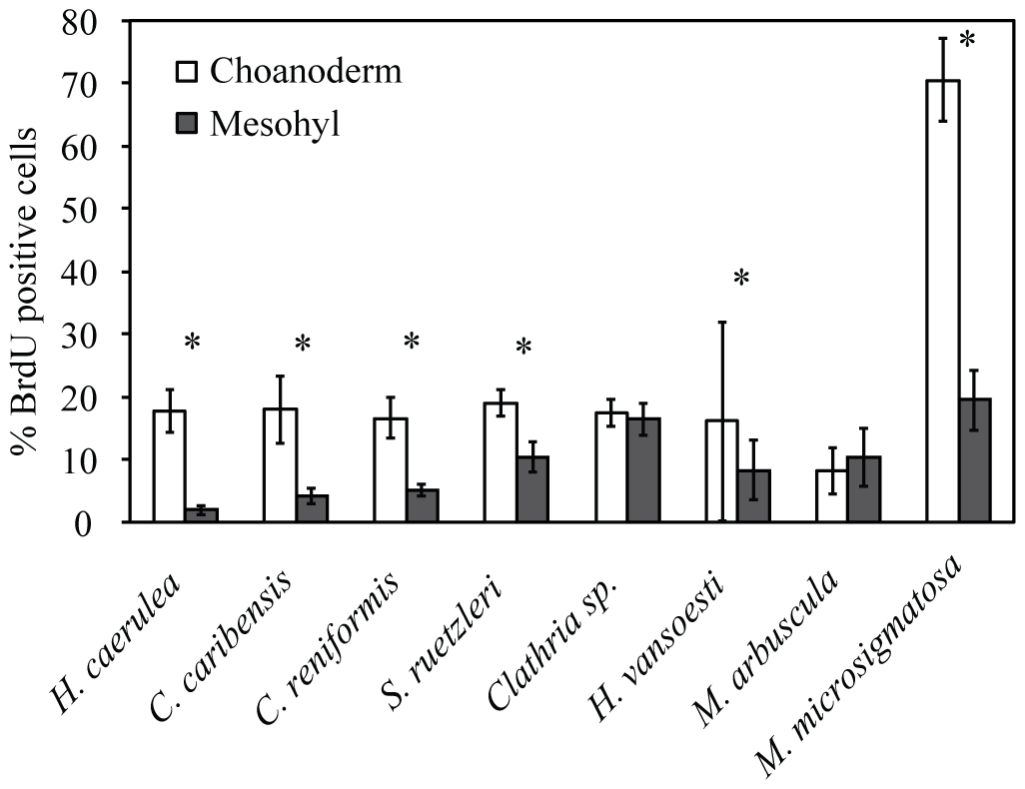
Cell proliferation in eight marine sponge species after continuous BrdU-labeling. Mean percentages (±SD) of BrdU-positive cells in the choanoderm (open bars) and mesohyl (solid bars) are shown. Significant differences between cell proliferation in the choanoderm versus mesohyl are indicated for each species (* p<0.001; 95%-CI are given in Table 2). All species were labeled with BrdU for 6 h except for *C. reniformis* which was labeled for 10 h.

**Figure 2:**
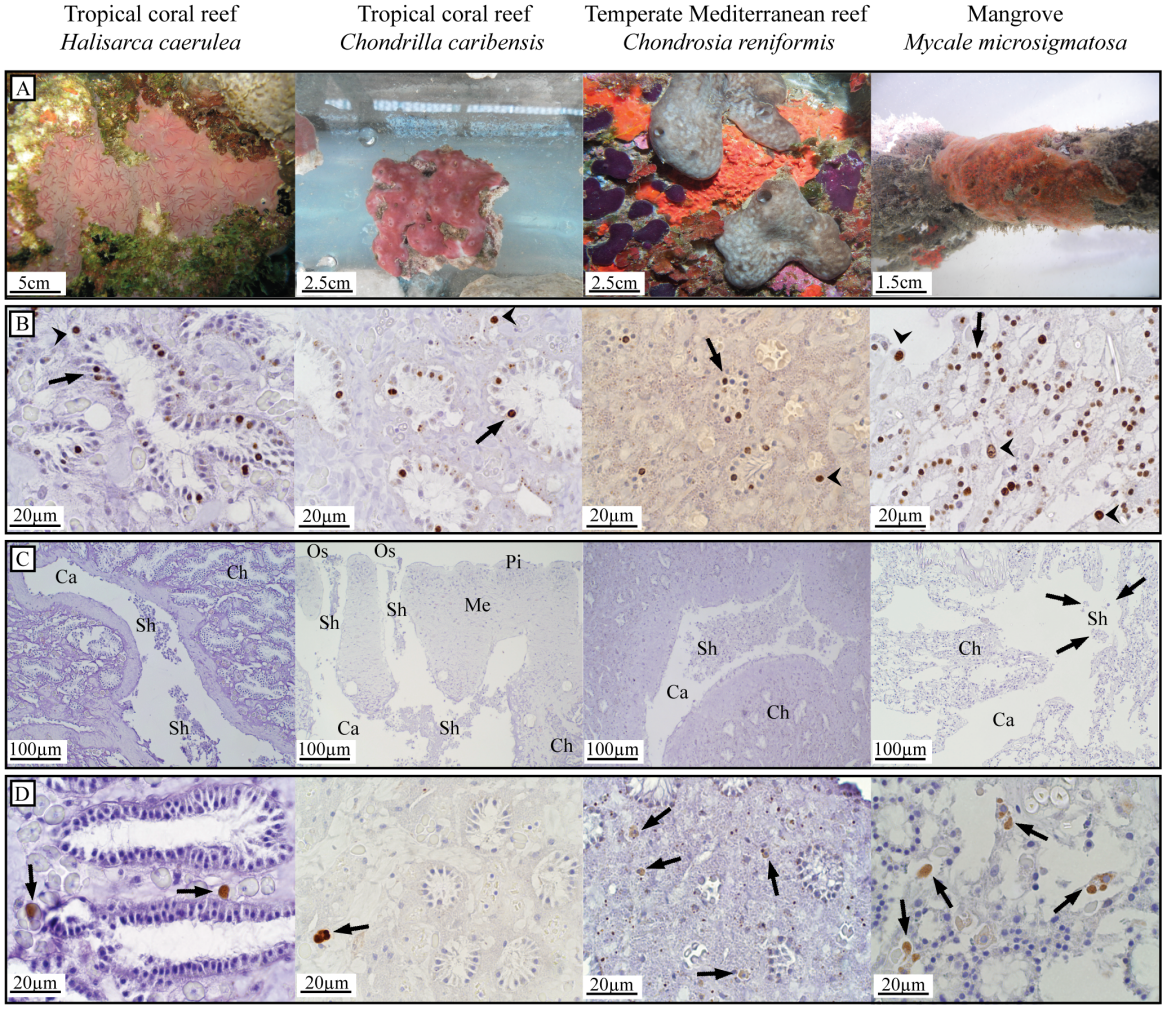
Cell proliferation and cell loss in a selection of four sponge species. Sponge species from three benthic ecosystems; tropical coral reef, temperate Mediterranean reef, and mangroves. (A) *In situ* (*H. caerulea, C. reniformis and M. microsigmatosa*) and *ex situ* (*C. caribensis*) photographs of test species. (B) BrdU-positive choanocytes (arrows) and mesohyl cells (arrowheads) of sponges BrdU-labeled for 6 h (10 h for *C. reniformis*) *in vivo* as a measure for proliferation. Areas of non-specific BrdU-labeling are occasionally seen in the cytoplasm of cells or extracellularly. (C) High amounts of cell shedding (Sh) in the lumen of excurrent canals (Ca) in specimens of *H. caerulea, C. caribensis* and *C. reniformis* sampled *in situ*. Choanocyte chambers (Ch), oscula (Os), the mesohyl (Me) and pinacoderm (Pi) are shown. Minor amounts of cell shedding (arrows) in the tropical mangrove sponge *M. microsigmatosa* sampled *in situ*. (D) Active caspase-3 activity of *in vivo* tissue was found in cells located in the mesohyl (arrows) resembling spherulous cells and, occasionally, archeocytes.

BrdU-positive choanocytes and mesohyl cells were observed in all species (Fig. 1, Fig. 2B, Fig. S1B, Table 1). The mouse intestine positive control tissue (Fig. S2A) and sponge tissue sections clearly showed BrdU-positive nuclei (brown-stained) and BrdU-negative nuclei (blue-stained), whereas only small amounts of non-specific BrdU-labeling were present in the cytoplasm or extracellularly (Fig. 2B, Fig. S1B). Negative controls (no primary antibody) showed no BrdU-positive cells (Fig. S2B). In general, choanocytes were more proliferative than mesohyl cells, and the effect varied with species (linear mixed model, p<0.001). There was a significant difference in the percentage of proliferating choanocytes versus mesohyl cells for six out of the eight species studied; *H. caerulea*, *C. caribensis*, *C. reniformis*, *S. ruetzleri*, *H. vansoesti* and *M. microsigmatosa* (Fig. 1, linear mixed model; mean differences and 95%-CI in Table 2). *Clathria* sp. and *M. arbuscula* showed no significant difference in the percentage of proliferating choanocytes versus mesohyl cells (Fig. 1, linear mixed model; mean differences and 95%-CI in Table 2).

**Table 1:** Cell proliferation (choanocytes and mesohyl cells), cell loss (shedding and detritus production), and microbial abundance categorization (HMA/LMA) in eight demosponge species (Porifera: Demospongia) from three benthic ecosystems (tropical coral reef, temperate Mediterranean reef and mangrove).

Species	*Halisarca caerulea*	*Chondrilla caribensis*	*Chondrosia reniformis*	*Scopalina ruetzleri*	*Clathria* sp.	*Haliclona vansoesti*	*Monanchora arbuscula*	*Mycale microsigmatosa*
Habitat	Tropical coral reef	Tropical coral reef	Temperate Mediterranean reef	Tropical coral reef	Tropical coral reef	Tropical coral reef	Tropical coral reef	Mangrove
% BrdU-positive choanocytes	17.6±3.3	18.0±5.4	16.6±3.2*	19.0±2.0	17.3±2.2	16.1±15.9	8.1±3.7	70.5±6.6
% BrdU-positivemesohyl cells	2.0±0.8	4.2±1.3	5.0±0.9*	10.4±2.4	16.4±2.5	8.3±4.8	10.3±4.7	19.5±4.9
Observed shedding	high	high	high	low	low	NA	low	low
Microbial abundance classification	L/HMA [56]	HMA	HMA [54]	LMA	LMA	LMA	LMA	LMA
Detritus production								
mg DW d^−1^	16.3±11.4	12.8±9.5	NA	15.0±8.2	NA	14.3±4.5	18.0±5.8	NA
% bodymass^−1^ d^−1^	15.1±6.7	2.5±2.2	NA	5.2±3.8	NA	5.6±3.4	18.0±5.7	NA

mean±SD are shown; NA: not applicable; * indicates 10 h BrdU labeling.

**Table 2:** Mean differences in the percentage of cell proliferation between the choanoderm and mesohyl (see Fig. 1 for percentages of cell proliferation in choanoderm and mesohyl).

	% Proliferation difference	
Species	mean	95%-CI
*H. caerulea*	15.7	11.98–19.43*
*C. caribensis*	13.8	10.03–17.48*
*C. reniformis*	10.5	7.45–15.61*
*S. ruetzleri*	8.6	4.84–12.29*
*Clathria* sp.	1.0	−2.87–4.79
*H. vansoesti*	7.9	6.84–5.62*
*M. arbuscula*	−1.3	−5.04–2.41
*M. microsigmatosa*	51.0	47.30–54.75*

The 95%-CI are estimated using a linear mixed model based on an F-test with Kenward-Roger approximation.

*  =  Significant difference in percentage proliferation between choanocytes and mesohyl cells.

Five out of seven sponge species had similar levels of choanocyte proliferation (mean±SD throughout) after labeling with BrdU for 6 h, ranging between 16.1% and 19.0%; *H. caerulea* (17.6±3.3%), *C. caribensis* (18.0±5.4%), *S. ruetzleri* (19.0±2.0%), and *Clathria* sp. (17.3±2.2%) and *H. vansoesti* (16.1±15.9%) (Table 1, Fig. 1, Fig. 2B, Fig. S1B). The tropical coral reef sponge, *M. arbuscula*, had lower levels of choanocyte proliferation compared to other species, with a mean of 8.1±3.7% (Table 1, Fig. 1, Fig. S1B). The highest percentage of BrdU-positive choanocytes (70.5±6.6%) was found in the mangrove species *M. microsigmatosa* (Table 1, Fig. 1, Fig. 2B). The tropical coral reef species *H. vansoesti* displayed a high variation in the amount of choanocyte proliferation ranging from as low as 2.8% to as high as 73.1% after 6 h of continuous labeling (Table 1, Fig. 1). This variability was found between different individuals and within tissue samples from the same individual. The Mediterranean species, *C. reniformis*, had 16.6±3.2% choanocyte proliferation after a 10 h BrdU-labeling (Table 1, Fig. 1, Fig. 2B). No difference was found in choanocyte proliferation between 6 h (17.6±3.3%) and 10 h (16.9±3.9%) of BrdU-labeling for *H. caerulea* (linear mixed model, p = 0.697). For all species, there was no significant difference in cell proliferation between tissue areas with and without an osculum (linear mixed model, p = 0.586). No notable change in shape or biomass increase was observed in sponges over time. No apparent relation was found between cell proliferation and microbial abundance classification of sponge species. Species with similar levels of choanocyte proliferation after 6 h of BrdU-labeling (between 16.1% and 19.0%), were either classified as HMA (*H. caerulea* and *C. caribensis*) or LMA (*S. ruetzleri* and *Clathria* sp.) (Table 1).

### Cell shedding

**Figure 3:**
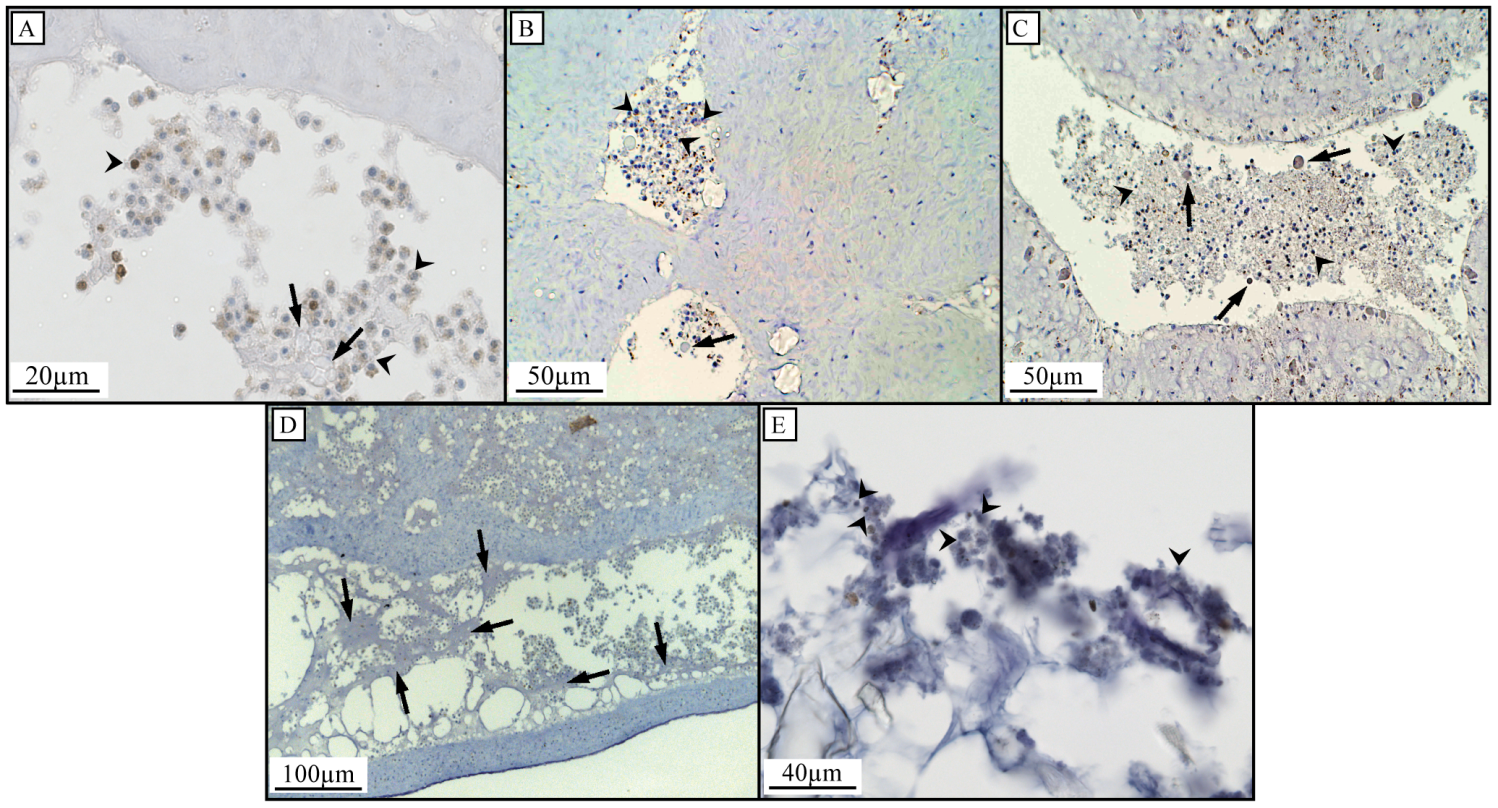
Histological investigations of cell shedding and identification of cellular debris in sponge-derived detritus. Arrowheads indicate shed choanocytes and arrows indicate shed spherulous cells in *H. caerulea* (A), *C. caribensis* (B) and *C. reniformis* (C). Shed cells in *H. caerulea* are present as mucal sheets (arrows) of cellular debris when close to the outflow openings (D). Light microscopy of detritus samples shows the presence of degraded cellular material, indicated by arrowheads (E).

Cell shedding was observed in tissue sections of all sponge species investigated, except *H. vansoesti*, for which cell shedding could not be determined (Table 1, Fig. 2C, Fig. S1C). Cellular debris was located inside the lumen of excurrent canals leading to the oscula (outflow openings) (Fig. 2C). BrdU-positive shed choanocytes (identified based on their size and shape) were found in all species in which cell shedding was observed, indicating the choanocyte origin of the shed cellular debris and fast cell turnover of choanocytes (within 6–10 h). In addition to choanocytes, cells resembling spherulous cells were also shed into the lumen of excurrent canals (Fig. 3A, Fig. 3B, Fig. 3C). Closer to the outflow opening, shed cells transform into mucal sheets of cellular debris (Fig. 3D). Light microscopy analysis of detritus samples indicate that sponge-derived cellular debris degrades further once shed into the ambient water (Fig. 3E).

**Figure 4:**
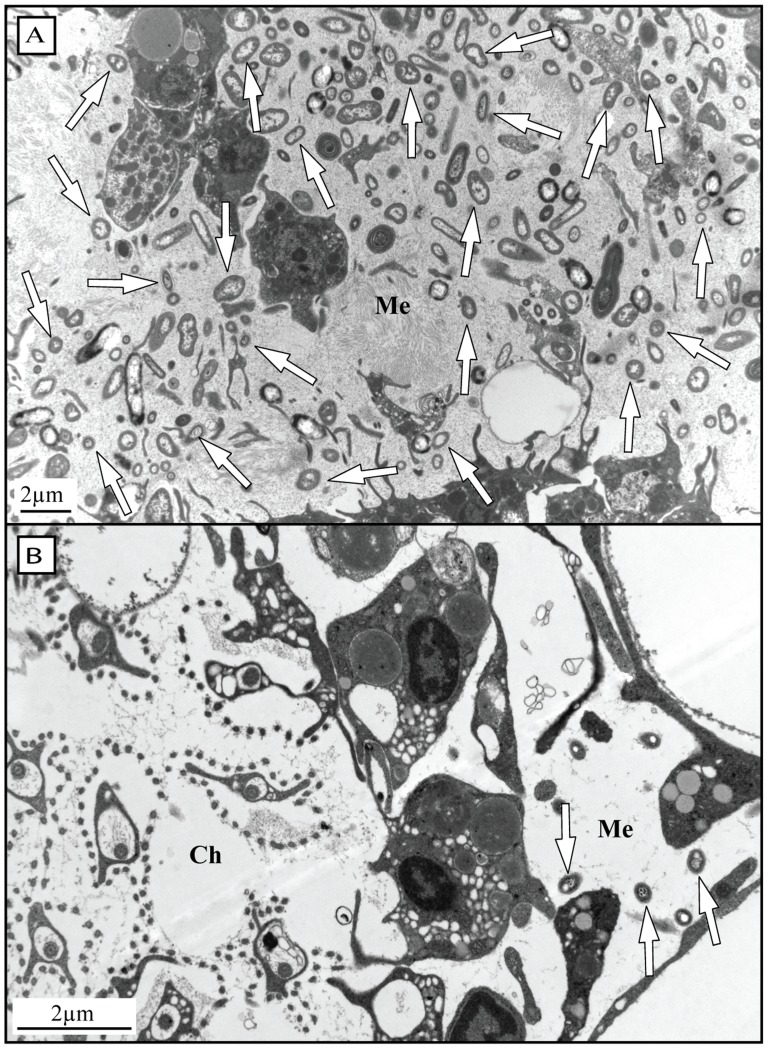
TEM images indicating microbial abundances in two sponge species. Arrows indicate selected bacterial symbionts. (A) Many associated microorganisms are present in the mesohyl (Me) of the high microbial abundance (HMA) species *C. caribensis*. (B) Choanoderm (Ch) and mesohyl (Me) of the low microbial abundance (LMA) species *M. microsigmatosa*. Only a few associated microorganisms are present.

In histological sections of each species, observed shedding was categorized as either ‘high’ or ‘low’ (Table 1). For example, high amounts of cell shedding were observed in *H. caerulea, C. caribensis* and *C. reniformis* (Fig. 2C), whereas only low amounts of cell shedding were observed in *M. microsigmatosa* (Fig. 2C), *S. ruetzleri*, *Clathria* sp. and *M. arbuscula* (Fig. S1C). No relation was found between the categorization of observed *in situ* cell shedding as high or low and the percentage of choanocyte proliferation measured *in vivo* (linear mixed model, p = 0.295). However, high amounts of cell shedding were only observed in species identified as HMA, whereas low amounts of shedding were only observed in species identified as LMA (Table 1). TEM images confirmed *C. caribensis* to be a HMA species (Fig. 4A) and *S. ruetzleri, H. vansoesti, M. arbuscula* and *M. microsigmatosa* (Fig. 4B) to be LMA species.

### Detritus production

**Figure 5:**
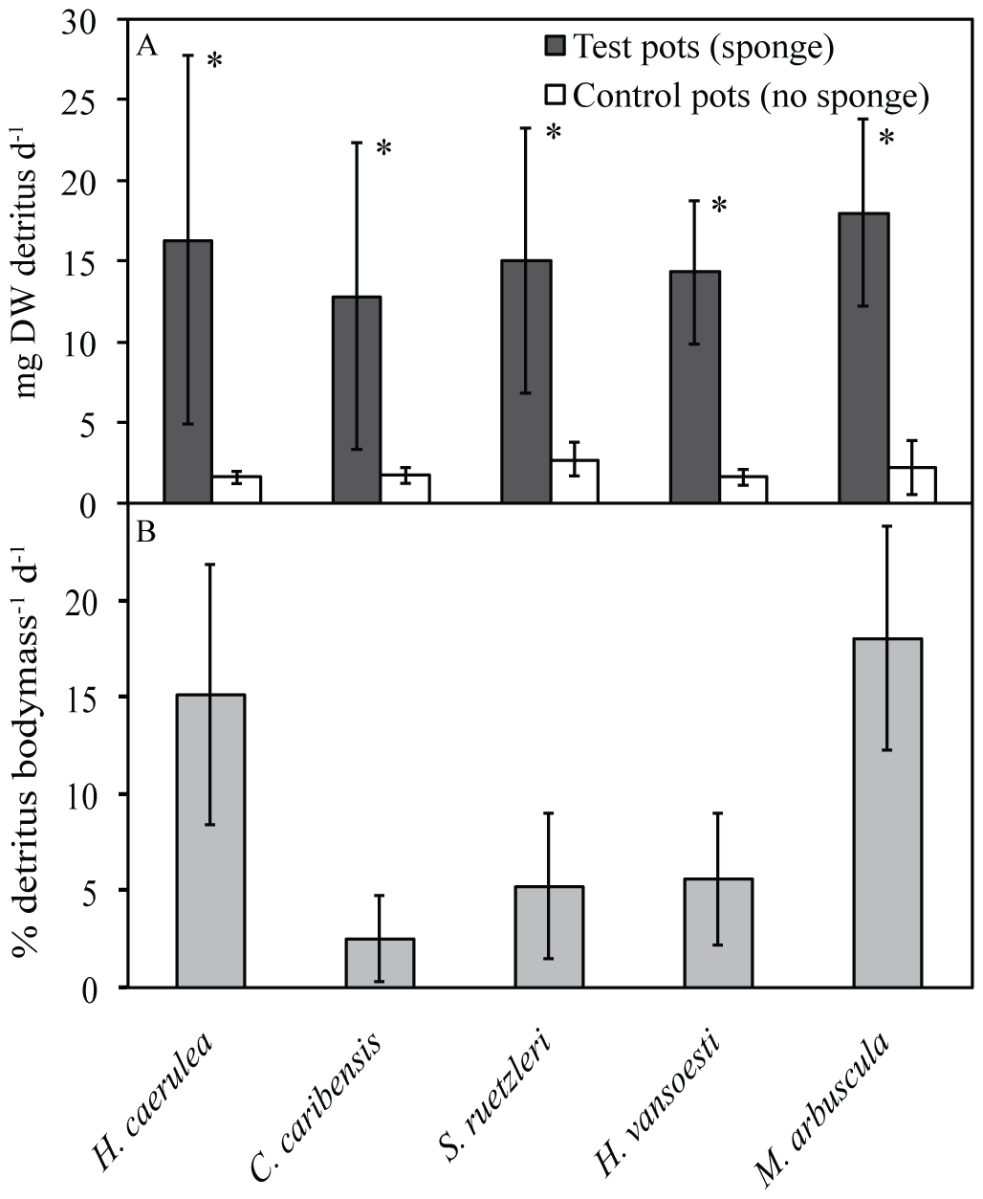
Daily detritus production of five tropical coral reef sponge species. (A) Daily detritus production (mg) and controls (mean±SD). Significant differences were found between detritus collected from pots containing sponge and control pots without sponge (* p<0.001). (B) Percentage bodyweight produced in detritus per day in five tropical coral reef species.

Daily detritus production (mean±SD throughout) in pots containing sponges (all species combined; 12.3±10.5 mg DW) was considerably higher than controls without sponge (2.0±0.7 mg DW) and the difference was highly significant (linear mixed model, p<0.001, Fig. 5A). After adjusting for controls, individual sponge species produced between 12.8±9.5 mg and 18.0±5.8 mg detritus per day (Table 1, Fig. 5A) amounting to an average daily detritus production per sponge biomass of 9.3±6.8% (range 2.5–18% detritus biomass^−1^ d^−1^, Table 1, Fig. 5B). There was no significant relationship between percentage detritus biomass^−1^ d^−1^ and observed cell shedding (categorized as high or low) per species (p = 0.337).

### Apoptosis

The human tonsil positive control tissue showed active caspase-3 positive cells with cytoplasmic staining (Fig. S2C). No active caspase-3 staining was found in negative controls (no primary antibody) (Fig. S2D). The number of active caspase-3-positive cells acted as a measure for apoptosis. Active caspase-3-positive cells were predominantly found in the mesohyl and accounted for <1% of total cells (Fig. 2D, Fig. S1D), and resembled spherulous cells and archeocytes based on their morphology, size and location in the tissue. Active caspase-3-positive choanocytes were rarely found. Specific staining was present in the cytoplasm of cells and often in inclusions of presumed spherulous cells with intact nuclei. No active caspase-3 positive cells were found in the tissue of *H. vansoesti*.

## Discussion

This study demonstrates that, similar to *H. caerulea* [6], tissue homeostasis in sponges under steady-state (non-growing) conditions occurs through cell turnover via high levels of cell proliferation (dominated by choanocytes) and cell shedding. This occurs in a variety of sponge species from different tropical and temperate benthic ecosystems. Understanding cellular processes of tissue homeostasis in sponges allows insight into the physiological state of sponges and mechanisms of sponge-mediated energy and nutrient transfer within benthic ecosystems.

### Cell proliferation

*C. caribensis, S. ruetzleri*, *Clathria* sp., and *H. vansoesti* all show similar percentages of choanocyte proliferation after 6 h of BrdU-labeling (between 16.1% and 19.0%) to that observed in *H. caerulea* (17.6%), although the variation in *H. vansoesti* was high. The two remaining species showed either consistently lower (*M. arbuscula*; 8.1%) or higher (*M. microsigmatosa*; 70.5%) percentages of choanocyte proliferation after 6 h. We were only able to measure cell proliferation after 10 h of BrdU-labeling in the Mediterranean species *C. reniformis*, and therefore it is difficult to compare the estimated rate of choanocyte proliferation (16.6% in 10 h) to the tropical species. The amount of choanocyte proliferation found in all species and the identification of the choanoderm as the predominant location of proliferating cells in the majority of species demonstrates that a high level of choanocyte proliferation is common in sponges and spans diverse ecosystems and sponge taxa. Estimates of choanocyte proliferation rates given here are considered high since, under steady-state conditions, the time to replace the number of choanocytes equivalent to the entire choanocyte compartment ranges from approximately 8.5 h in *M. microsigmatosa* to 74 h in *M. arbuscula*. The slowest rate (74 h or 3 days) is comparable to known high turnover rates in cell populations of other metazoans, such as the endodermal gland cells of *Hydra attenuata* (3.5 days) [34] and the epithelium of the mammalian gastrointestinal tract (3–5 days) [35]–[37]. Proliferation rates presented here may be underestimated if the sponges investigated would have cell cycle durations considerably shorter than 6 h. To determine the cell cycle duration and growth fraction of choanocytes in each species, cell cycle analyses should be carried out by labeling sponges with BrdU at multiple time points [6], [31].

In this study, we found a considerably lower percentage (17.6±3.3%) of choanocyte proliferation in *H. caerulea* after 6 h of continuous BrdU-labeling compared with the percentage of proliferation (46.6±2.6%) previously found *in vivo* and *in situ* for the same species [6]. Suboptimal nutritional conditions in the aquaria compared to the previous study are arguably the cause of this difference. Food intake is one of the most important stimuli influencing homeostatic cell proliferation in uptake systems such as the mammalian gastrointestinal tract [38], [39]. Proliferation rates of mouse and rat intestinal epithelial cells decrease during starvation and subsequently increase after re-feeding [39], [40]. Cell renewal is an energetically-costly process and a reduction in proliferation is an adaptive mechanism enabling an organism to survive during periods of reduced food intake. During fieldwork campaigns for this study, bacterial concentrations (as a proxy for food concentration) in the tropical aquaria had decreased to only one-third of ambient reef water concentrations due to long-term bio-fouling of sponges and other filter feeders in the water inlet pipes. Reduced cell proliferation following starvation has been found to be due to a prolonged cell cycle, e.g. [40], or a decrease in the number of actively proliferating cells, e.g. [41]. No difference was found between the percentage of proliferative choanocytes after 6 h and 10 h of BrdU-labeling in *H. caerulea*, suggesting that the choanocyte cell population had reached its growth fraction. Therefore, we hypothesize that for *H. caerulea*, the growth fraction becomes reduced with a decline in food availability. The relationship between nutrient concentration and cell proliferation should be investigated in future work to confirm this hypothesis. We conclude that the percentages of cell proliferation and subsequent amount of cell loss presented here may be underestimated compared to *in situ* physiological conditions, with one exception: the mangrove species, *M. microsigmatosa*, had extremely high proliferative activity in both choanocytes (70.5±6.6%) and mesohyl cells (19.5±4.9%) compared to other species. This may have been caused by additional nutrients obtained directly from the root substrate. Mangrove sponges have a facultative mutualistic relationship with the roots they overgrow, directly using carbon derived from roots as an energy source [42], [43]. When experimentally attached to mangrove prop roots, growth rates of *Tedania ignis* and *Haliclona implexiformis* increased 1.4–10 times compared to sponges grown on PVC tubes due to nutrient exchange between the roots and the sponges [43]. A direct supply of nutrients from the root substrate may have caused *M. microsigmatosa* to be less affected by sub-optimal aquarium conditions during the experimental period compared to the reef species, and thus facilitated higher levels of cell proliferation. It is unknown whether an extremely high level of cell proliferation is a characteristic intrinsically specific to this species or a characteristic of all sponges inhabiting mangroves.

We assumed that sponges were in steady-state conditions based on observations that they only changed marginally in shape or size during the experimental period (1–4 weeks). If proliferation would have been allocated to growth (biomass increase), the levels of cell proliferation measured would indicate biomass doubling rates in terms of days. However, we cannot fully exclude that some sponges, or parts of the sponge, may have been in an alternative physiological state such as tissue regression [44], tissue regeneration, e.g. [12], [45], or somatic growth, e.g. [46], [47]. Tissue regeneration and somatic growth may influence homeostatic cell turnover due to competing demands on limited energy resources [48]. An alternative physiological state occurring in some parts of a sponge may explain the high variation (2.8–73.1%) in choanocyte proliferation in *H. vansoesti* and may also be the cause of the low choanocyte proliferation observed for *M. arbuscula*. Choanocytes were not the predominant population of proliferating cells in *M. arbuscula* and there was no difference in proliferation between choanocytes and mesohyl cells. To gain insight into the relationship between the physiological state of a sponge and cell proliferation, the cell kinetics of both choanocytes and other cell types, such as mesohyl cells, should be further examined.

Proliferative cells located in the mesohyl presumably consist primarily of archeocytes based on their size, shape, location and mitotic activity [46]. Choanocytes and archeocytes are both known to be the predominant populations of proliferating cells in sponges [6], [49], [50]. The division of archeocytes may be related to self-renewal of the choanocyte population to replace cells lost due to shedding during steady-state tissue homeostasis. Choanocyte self-renewal may either be the result of the division of archeocytes committed to the choanocyte lineage or choanocyte division [51]. Proliferation in archeocytes may also correspond to somatic growth [46] or regenerative growth [48], both of which require the division of archeocytes for cell differentiation and for self-renewal of the archeocyte cell population [50].

### Cell loss: shedding

Sponges require a mechanism of cell loss to counterbalance the high levels of cell proliferation under non-growing conditions. The presence of a large number of choanocytes (including BrdU-positive choanocytes) in the canals leading to the outflow openings of the sponges provides evidence that cell shedding is the principle mechanism of cell loss to maintain homeostasis in the choanocyte compartment. Aside from choanocytes, we found that cells from the mesohyl resembling spherulous cells were also shed. This corroborates with previous studies on *H. caerulea* [6], [52] and TEM studies describing shed cells (including various mesohyl cell types) in the excurrent canals of other sponge species (Maldonado, unpublished data). Cell shedding could not be analyzed in *H. vansoesti* due to its loose tissue morphology, making it difficult to distinguish between cells within the mesohyl and shed cells in histological sections.

The amount of shed cells observed in histological sections is not always as much as expected based on cell proliferation rates. For example, *S. ruetzleri*, *Clathria* sp. and *M. microsigmatosa* displayed substantial proliferation in choanocytes but only small amounts of cell shedding were observed in their tissue sections compared to *H. caerulea*, *C. caribensis* and *C. reniformis*. Interestingly, the amount of observed shedding in histological sections of sponges seems to be related to their microbial abundances and may be influenced by the residence time of cellular debris within the canal system. Water pumping rates and tissue densities of sponges have been found to be related to the abundance of sponge-associated microbes. High microbial abundance (HMA) species have more dense collagenous tissues and slower pumping rates than their low microbial abundance (LMA) counterparts, which generally have loose tissue morphologies and high pumping rates [28], [53]. All species in which low amounts of cell shedding were observed have been identified as LMA sponges and species in which high amounts of cell shedding were observed are HMA sponges. Slow pumping rates may cause longer residence times of cellular debris in the canals, which subsequently may be more easily visualized in tissue sections. Higher pumping rates in the well-irrigated LMA species may cause the shed material to be quickly removed from the sponge tissue with the water flow. Oscular outflow measurements are required to confirm this hypothesis.

### Cell loss: detritus production

We attempted to directly link the process of cell shedding and the production of detritus in sponges. Isotope tracer studies for *H. caerulea*, *C. caribensis*, *S. ruetzleri* and *H. vansoesti* have shown that these species convert assimilated DO^13^C and DO^15^N into detrital PO^13^C (11–24% in 3 h) and PO^15^N (18–36% in 3 h) respectively [3]. Although all sponge species investigated here produced significant amounts of detritus, there was high variability in the amount of detritus produced, and no apparent relation between detritus production and the amount of cell shedding observed in tissue sections per species. This may be due to variation in the composition of detritus between species and the methodology used. It was impossible in practical terms to be certain that all sponge-produced detritus was captured and collected in the experimental pots. Lighter detrital particles may have been lost in the flow-through aquarium set-up. Detritus has been described as being derived from feces, mucus and other indigestible waste (concentrated filtered non-digested particles) [4], [22]–[25], and therefore detrital composition is likely to be species dependent. Furthermore, due to the degraded nature of the detritus (only cell remnants could be identified), it was impossible to quantify the amount and type of cells within the detritus. Therefore, at present we cannot predict to what extent shed choanocytes, along with shed mesohyl cells, contribute to detritus production.

### Cell loss: apoptosis

Apoptosis is clearly not the principal mechanism by which cells are lost in order to maintain tissue homeostasis in the choanocyte compartment of sponges under steady-state conditions. In all species, apoptotic activity was largely absent in the proliferative choanocyte compartment and only found in a few cells, morphologically resembling spherulous cells or occasionally resembling archeocytes, as previously found for *H. caerulea* [6]. Many of the active caspase-3 positive cells had intact nuclei. These cells may have phagocytized apoptotic bodies or may be in the early stages of apoptosis in which nuclear fragmentation has not yet occurred. Spherulous cells are expelled into excurrent canals [6], [52] and it has been suggested that apoptosis acts as part of the waste control system in sponges rather than acting to maintain tissue homeostasis [6]. Although the positive and negative controls provide evidence for specific active caspase-3 staining, a western blot is required to validate the specificity of the anti-active caspase-3 antibody in sponge tissue.

### Cell turnover and the sponge loop

Recently, one of the major energy and nutrient cycling pathways occurring on coral reefs, the so-called ‘sponge loop’, has been found to depend on three prerequisites: (1) the uptake of DOM by sponges, (2) the conversion of DOM into detritus and (3) the uptake of sponge-derived detritus by detritivores [3]. Cell turnover (cell proliferation and cell shedding leading to detritus production) is proposed to be the main underlying mechanism involved in the transfer of DOM to higher trophic levels by sponges [3]. In this study, we found that all four sponge species investigated by De Goeij and colleagues [3] exhibit high levels of cell proliferation along with detritus production. Additionally, cell turnover, predominantly through choanocyte proliferation and cell shedding, has been found in sponges from other sponge-dominated benthic ecosystems, namely mangroves and temperate Mediterranean reefs. The ability of the Mediterranean sponge *C. reniformis* [54] and the mangrove sponge *Niphates erecta* [55] to take up DOM, another prerequisite of a sponge loop pathway, suggests that resource cycling within these ecosystems may be controlled by a cell turnover-driven sponge loop pathway. Cellular processes of tissue homeostasis provide insight into how sponges may convert assimilated (together with concentrated indigested) organic matter into detritus, which serves as a food source for higher trophic levels.

## Supporting Information

Figure S1 **Cell proliferation and cell loss in four species of tropical coral reef sponge.** (A) *In situ* (*S. ruetzleri, H. vansoesti*) and *ex situ* (*Clathria* sp., *M. arbuscula*) photographs of test species. (B) BrdU-positive choanocytes (arrows) and mesohyl cells (arrowheads) of sponges BrdU-labeled for 6 h *in vivo* as measure of cell proliferation. Areas of non-specific BrdU-labeling can occasionally be seen in the cytoplasm of cells or extracellularly. (C) Minor amounts of cell shedding (Sh, arrows) shown for *S. ruetzleri, Clathria* sp. and *M. arbuscula*. No shedding could be identified in histological sections of *H. vansoesti*. Choanocyte chambers (Ch), the mesohyl (Me) and excurrent canals (Ca) are shown. (D) Active caspase-3 activity of *in vivo* tissue was confined to cells located in the mesohyl (arrows). No active caspase-3 positive cells were found in the tissue of *H. vansoesti*.
(TIF)

Figure S2 **Positive and negative controls for BrdU and active caspase-3 immunohistochemistry.** (A) BrdU-labeled mouse intestine positive control tissue. Arrows indicate BrdU-positive intestinal epithelial cells (brown-stained). (B) BrdU-labeled mouse intestine negative control tissue (no primary anti-BrdU antibody) showed no BrdU-positive cells. (C) Active caspase-3 positive cells (brown-stained, indicated by arrows) in human tonsil positive control tissue. (D) Human tonsil negative control (no primary anti caspase-3 antibody) showed no active caspase-3 positive cells.
(TIF)
