# Supplementary figures and images for: Cell Turnover and Detritus Production in Marine Sponges from Tropical and Temperate Benthic Ecosystems
Source: PLoS One. 2014 Oct 7;9(10):e109486. doi: 10.1371/journal.pone.0109486 (PMC4188633; doi:10.1371/journal.pone.0109486)

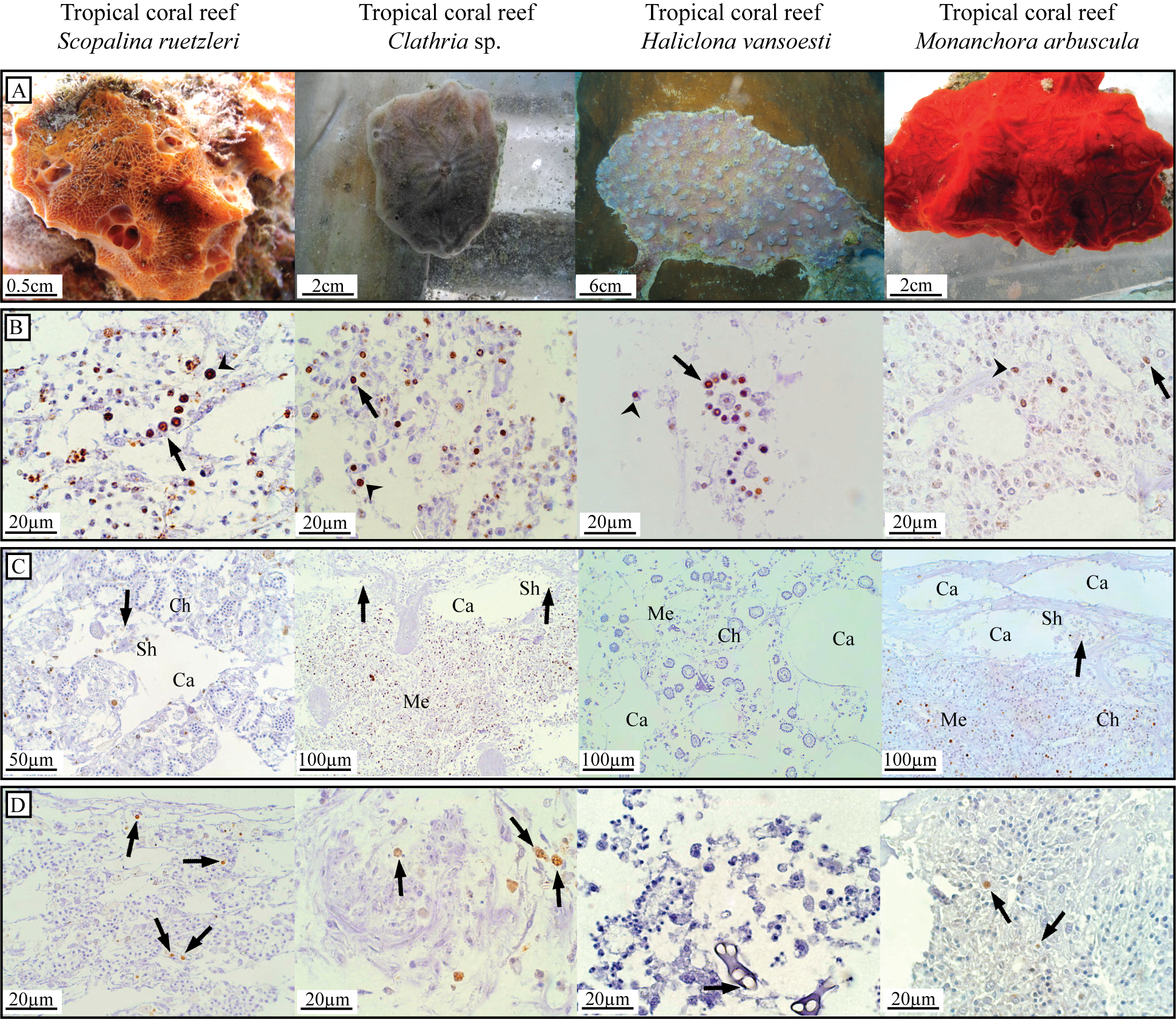

Supplement: Figure S1 — Cell proliferation and cell loss in four species of tropical coral reef sponge. (A) In situ (S. ruetzleri, H. vansoesti) and ex situ (Clathria sp., M. arbuscula) photographs of test species. (B) BrdU-positive choanocytes (arrows) and mesohyl cells (arrowheads) of sponges BrdU-labeled for 6 h in vivo as measure of cell proliferation. Areas of non-specific BrdU-labeling can occasionally be seen in the cytoplasm of cells or extracellularly. (C) Minor amounts of cell shedding (Sh, arrows) shown for S. ruetzleri, Clathria sp. and M. arbuscula. No shedding could be identified in histological sections of H. vansoesti. Choanocyte chambers (Ch), the mesohyl (Me) and excurrent canals (Ca) are shown. (D) Active caspase-3 activity of in vivo tissue was confined to cells located in the mesohyl (arrows). No active caspase-3 positive cells were found in the tissue of H. vansoesti. (TIF) [file pone.0109486.s001.tif]

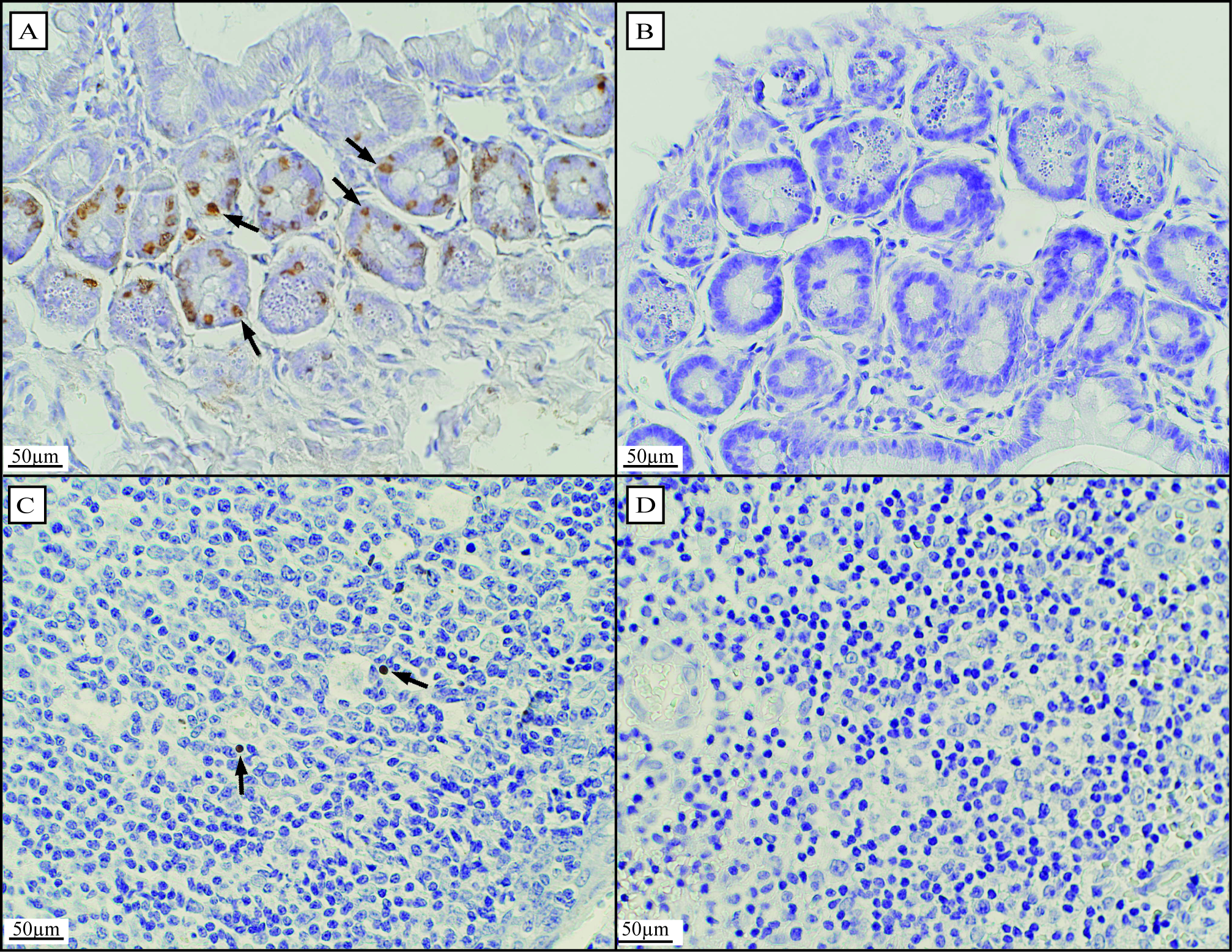

Supplement: Figure S2 — Positive and negative controls for BrdU and active caspase-3 immunohistochemistry. (A) BrdU-labeled mouse intestine positive control tissue. Arrows indicate BrdU-positive intestinal epithelial cells (brown-stained). (B) BrdU-labeled mouse intestine negative control tissue (no primary anti-BrdU antibody) showed no BrdU-positive cells. (C) Active caspase-3 positive cells (brown-stained, indicated by arrows) in human tonsil positive control tissue. (D) Human tonsil negative control (no primary anti caspase-3 antibody) showed no active caspase-3 positive cells. (TIF) [file pone.0109486.s002.tif]
